# Covering Nasometer Microphones with Plastic Wrap for Infection Control Increases Retest Variability of Nasalance Scores

**DOI:** 10.1177/10556656211051582

**Published:** 2021-11-23

**Authors:** Tim Bressmann

**Affiliations:** 1 Department of Speech-Language Pathology, University of Toronto, Toronto, Ontario, Canada

**Keywords:** acoustics, speech production, nasality, resonance, aerodynamics

## Abstract

The Nasometer is a popular instrument for the acoustic assessment of nasality. In light of the currently ongoing COVID-19 global pandemic, clinicians may have wondered about the infection control procedures for the Nasometer. The current research investigated whether nasalance scores are affected if the Nasometer 6450 microphone casings are covered with a material such as rolled polyvinyl chloride household wrap. For the experiment, pre-recorded sound files from two speakers were played back through a set of small loudspeakers. Nasalance scores from two baselines and three wrap cover conditions were compared. While there was no statistically significant condition effect in a repeated-measures analysis of variance, the within-condition cumulative differences in nasalance scores were 2 for the initial baseline, 42 for wrap cover 1, 24 for wrap cover 2, 78 for wrap cover 3, and 8 for the final baseline. Mean differences between the wrap cover and the baseline conditions were 8.2 to 15.3 times larger, and cumulative differences were 8.3 to 16.6 times larger than between the two baselines. Based on the higher cumulative and mean differences observed, clinicians should not cover Nasometer microphones with household wrap as this increases variability of nasalance scores. Since there is evidence that the COVID-19 virus can survive for some time on metal surfaces, clinicians should be mindful of the fact that the Nasometer microphone housings can only be cleaned superficially and should be handled with gloves to minimize any possible risk of touch transfer of pathogens to the next speaker or the clinician.

## Introduction

The Nasometer (Pentax Medical, Montvale, NJ) is a popular instrument for the quantitative acoustic assessment of nasality disorders in different clinical populations, including individuals with cleft palate. To assess whether too much or too little sound is emanating from a speaker's nose, a sound separation plate is positioned on the speaker's prolabium, and microphones on the top and bottom of the baffle plate are used to measure sound pressure levels (SPLs; Fletcher, 1978; Kummer, 2020). The nasal and oral sound pressure levels are then used to calculate the mean nasalance score, based on the formula % Nasalance = (nasal SPL/(nasal SPL + oral SPL)) × 100, so that nasalance expresses the proportion of the nasal signal to the total speech signal (Fletcher et al., 1974).

According to the widely used Spaulding classification system (Spaulding, 1957), the Nasometer should require level 2 safety procedures because it comes into contact with unbroken skin (the speaker's prolabium). The Nasometer baffle plate is made from metal, with a silicone tube slid onto the edge for more comfort and to protect the teeth while the Nasometer headset is positioned. The silicone tubing and the metal separator plate are disinfected using a rubbing alcohol such as isopropyl. The microphones, which are positioned about 4 cm away from the speaker's face, are held in fibreglass housings, which are attached to the metal baffle plate with a screw. Since the microphones contain electric components and wiring, they can only be wiped lightly with alcohol.

**Figure 1.**
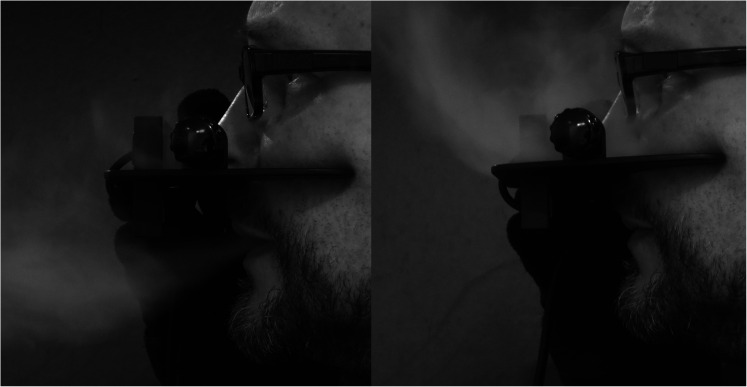
Exhalation of vapour against a nasometer baffle plate during the production of sustained /a/ (left) and sustained /m/ (right).

In light of the currently ongoing coronavirus disease 2019 (COVID-19) global pandemic, clinicians have wondered about the infection control procedures for the Nasometer. Figure 1 shows still images of vapour exhalation onto the Nasometer baffle plate for sustained /a/ and /m/ sounds. Research has shown that the COVID-19 virus can still be found on metal such as stainless steel for as long as 72 h after application (van Doremalen et al., 2020). Surface contamination entails a risk of possible touch transfer to the next speaker or the clinician (Marquès and Domingo, 2021). While the Nasometer separator plate can be cleaned using alcohol, hydroxy peroxide or similar products, it is conceivable that small amounts of virus may remain on the microphone assembly.

A hygiene practice that can be used in addition to cleaning and disinfection is physical shielding with a barrier material such as plastic wrap (Jefferies et al., 1991). Commonly available materials such as rolled polyvinyl chloride household wrap can be used to prevent contamination of exposed equipment that is otherwise difficult to wash and sterilize, such as computer keyboards (Chen, Pradhan and Xue, 2020).

Any barrier material between a sound source and a microphone membrane will affect the microphone's response, for example when a foam cover is used to reduce wind noise or air pressure spikes from plosive consonants. Recent research on the effect of face masks on speech has demonstrated acoustic dampening effects especially on higher frequencies. Corey et al. (2020) found that frequencies above 1000 Hz were attenuated with various face masks. Magee et al. (2020) also observed attenuating effects of face masks on higher frequency components of speech but demonstrated that acoustic voice quality measures such as the cepstrum and the harmonics to noise ratio could still be measured with accuracy. Nasalance scores are calculated based on sound pressure level measurements in a signal that is bandpass filtered around a 500 Hz centre frequency, i.e., the low frequency components of the speech signal (Kummer, 2020). Such low frequencies transmit well through thin barrier materials such as plastic wrap. Therefore, it was of interest to test whether the same nasalance scores would be obtained if the Nasometer microphone casings were covered with a single layer of plastic wrap, compered to the normal recording condition.

The research was explorative and not guided by a specific hypothesis. Differences between nasalance scores between covered and uncovered conditions as well as variability of nasalance scores served as outcome measures.

## Methods

In the present study, the Nasometer was tested with single layers of Glad Press ‘n Seal rolled polyvinyl chloride household wrap (Clorox, Oakland, CA) over the microphones. Press ‘n Seal is textured on one side and has a light adhesive applied to the other side. The combination of the adhesive and the textured surface makes the material stick to itself as well as to the items to be wrapped. The material is air- and watertight. Among the range of readily available household wraps, it appeared to be the most obvious choice and the most convenient material to wrap the Nasometer headphones for infection control purposes.

To create test recordings, a Nasometer 6450 headset was connected to a Tascam DR-05 digital recorder (TEAC, Tokyo, Japan). Two typical adult speakers (one female, one male) were recorded producing two repetitions each of the non-nasal sentences “Look at this book with us. It's a story about a zoo,” the nasal sentence “Mama made some lemon jam” (Fletcher, 1978), and the phonetically mixed sentence “The rainbow is a division of white light into many beautiful colours” (Fairbanks, 1960), resulting in 12 test stimuli. The recordings were downloaded to a computer. For the experiment, the sound files were played back through a set of two small loudspeakers (iHM78; iHome, Rahway, NJ). Human speakers show variability in their nasalance scores when repeating the same stimuli over time (De Boer and Bressmann, 2014), so it would have been difficult to separate test-retest differences for human speakers from the effect of the household wrap on nasalance scores. The loudspeakers' frequency responses had been characterized in detail in a previous research study (De Boer and Bressmann, 2014).

**Figure 2.**
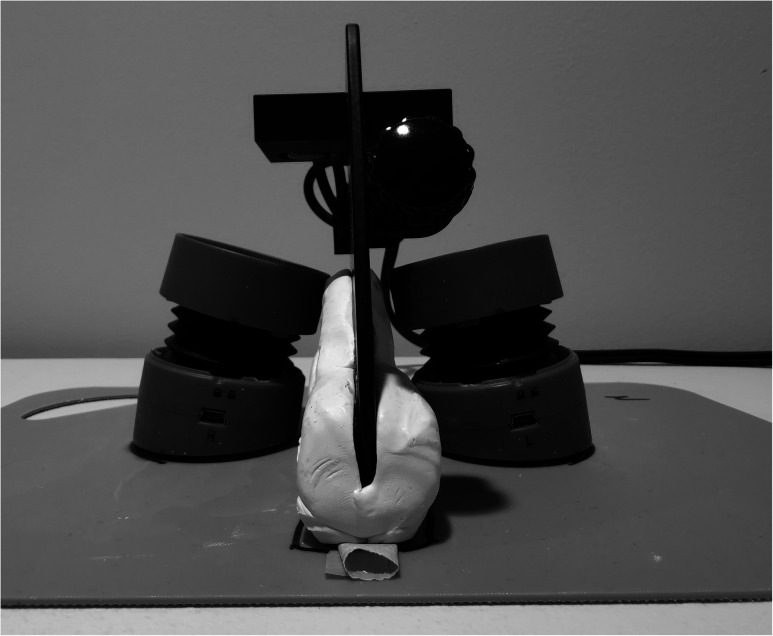
Test setup with nasometer holder and loudspeakers. Loudspeaker cables were removed for the photo.

A Nasometer 6450 was used for the experiment. The Nasometer software was run on a Dell Vostro 1000 laptop (Dell Canada, Toronto, ON). A holder for the Nasometer sound separation plate was made from Sculpey molding clay (Polyform Products, Elk Grove Village, IL). The Nasometer separation plate was positioned vertically in the holder, and the two loudspeakers were positioned so that the centres of the loudspeaker membranes were 3 cm away from the nasal and oral microphones, respectively (Figure 2). When the pre-recorded stereo soundfiles were played back, the nasal signal was played through the left channel loudspeaker and recorded with the nasal microphone of the Nasometer. The oral signal was played through the right channel loudspeaker and recorded with the oral microphone. All recordings were made in a quiet room.

**Figure 3.**
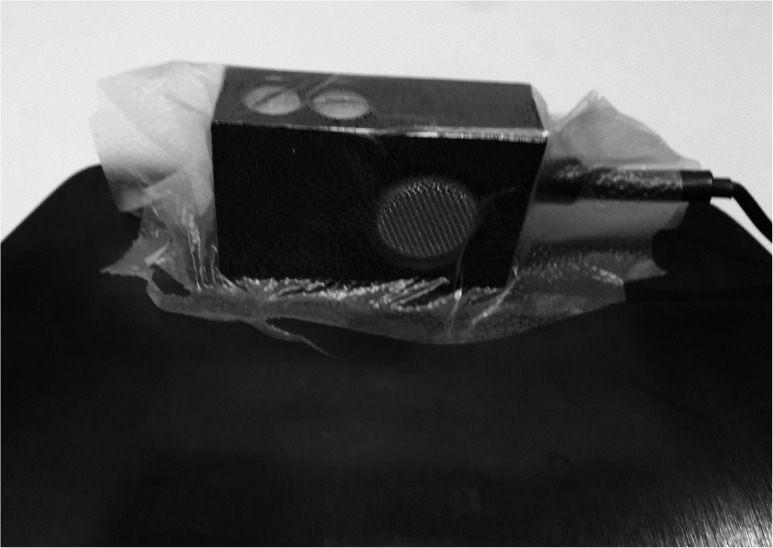
Nasometer microphone covered with household wrap.

The Nasometer was calibrated, and five sets of recordings were made. In the first baseline, each of the 12 test stimuli were recorded 3 times, so that 36 nasalance scores were obtained. The Nasometer plate was then removed from the holder and the two microphones were each covered with a single piece (8 cm by 8 cm) of household wrap as shown in Figure 3. The Nasometer separation plate headset was then placed back into the holder, and the 12 test sentences were re-recorded 3 times. This step was repeated 2 more times to remove the wrap and re-wrap the microphones with new pieces of Press ‘n Seal. This resulted in 3 sets of recordings with wrap-covered microphones. As the last step, a final baseline recording was made with uncovered microphones.

Outcome measures were mean nasalance scores and absolute differences between repetitions and different conditions. The 72 nasalance scores from the two baselines and the 108 nasalance scores from the three recordings with the wrap-covered microphones were analyzed with a repeated-measures analysis of variance. Differences between repetitions and conditions were analyzed descriptively.

## Results

Table 1 shows the mean nasalance scores and standard deviations for the recordings with and without wrap on the microphones. A repeated-measures analysis of variance was calculated with the Number Cruncher Statistical Software 8 (NCSS, Kaysville, UT), with the condition and the speech stimuli (non-nasal, mixed, nasal) as the within-subject effects. The analysis showed significantly higher nasalance scores for the female participant compared to the male speaker (df = 1, 150; *F* = 3033.72; *P *< 0.01). There was no significant effect for condition. There were significant differences for the 3 different speech stimuli (df = 2, 150; *F* = 200.3; *P* < 0.01). Bonferroni post-hoc tests showed that all 3 stimuli differed significantly from each other, with the highest nasalance scores for the nasal sentence and the lowest for the non-nasal sentences (all contrasts *P *< 0.05).

**Table 1.** Mean Nasalance Scores, Standard Deviations, and Cumulative Absolute Differences Between the Repeated Recordings

Item	Initial baseline	Wrap cover 1	Wrap cover 2	Wrap cover 3	Final baseline
Zoo (f)	24.0	22.7	29.2	27.7	24.5
	SD 1.1	SD 1.6	SD 1.3	SD 1.9	SD 1.6
	0	6	2	16	0
Zoo (m)	15.0	8.5	10.2	18.2	15.0
	SD 0.0	SD 0.5	SD 0.4	SD 1.7	SD 0.0
	0	4	2	10	0
Mama (f)	66.8	62	66.7	52.3	67.3
	SD 0.4	SD 0.9	SD 0.8	SD 4.0	SD 0.5
	2	8	2	20	4
Mama (m)	59.5	52	51.3	43.5	60.5
	SD 0.5	SD 1.4	SD 1.0	SD 1.9	SD 0.5
	0	8	6	8	0
Rainbow (f)	51.5	47.3	54	45.3	52.3
	SD 2.7	SD 2.1	SD 1.9	SD 2.0	SD 2.6
	0	8	4	16	2
Rainbow (m)	40.5	32	32.5	28.5	41.2
	SD 0.5	SD 0.9	SD 1.0	1.0	SD 0.4
	0	8	8	8	2
Sum of cumulative absolute differences	2	42	24	78	8

Data are reported for the female (f) and male (m) speakers for the different conditions with and without wrap covers.

In each of the experimental conditions, each of the 12 sound files (2 repetitions of 3 sentences by 2 speakers) was played back through the loudspeakers and re-recorded through the Nasometer headset 3 times. To assess variability between these nasalance scores of the repeated stimuli, the absolute differences in nasalance between the repetitions of the same stimuli were added up. For example, one such contrast were the 3 repetitions of the first recording of the Zoo sentences spoken by the female speaker. The sums of the within-condition cumulative differences in nasalance scores were 2 for the initial baseline, 42 for wrap cover 1, 24 for wrap cover 2, 78 for wrap cover 3, and 8 for the final baseline.

In a final step, the mean and the cumulative absolute differences between the mean nasalance scores for the three sets of stimuli (non-nasal, nasal and mixed) were calculated between the two baselines and the 3 wrap cover conditions. Table 2 shows the results. The differences were smallest between the two baselines (mean difference 0.6, cumulative difference 7). In comparison to the differences between the two baselines, the mean differences between the wrap cover and the baseline conditions were 8.2 to 15.3 times larger, and the cumulative differences were 8.3 to 16.6 times larger.

**Table 2.** Average and Cumulative Differences in Mean Nasalance Between Conditions.

Comparison	Mean nasalance difference	Cumulative nasalance difference
Baseline 1 vs. Baseline 2	0.6	7
Wrap cover 1 vs. Baseline 1	5.5	66
Wrap cover 1 vs. Baseline 2	6	73
Wrap cover 2 vs. Baseline 1	4.9	58
Wrap cover 2 vs. Baseline 2	4.9	59
Wrap cover 3 vs. Baseline 1	9.2	111
Wrap cover 3 vs. Baseline 2	9.7	116

## Discussion

The present study investigated the effect that covering the Nasometer 6450 microphones with thin layers of household wrap had on nasalance scores. Since human speakers show variability in speech and in their nasality levels over time (De Boer and Bressmann, 2014), the test signals were played from loudspeakers. While this was not a natural speech signal, it nevertheless allowed for consistent measurement of nasalance levels to accurately assess the effect of the household wrap on nasalance scores and their variability. This approach appeared to work well for the task, even if the nasalance scores measured from the loudspeakers may have differed, had the stimuli been measured directly from the human speakers.

Visual inspection of the results in Table 1 showed that the nasalance scores for the initial and final baselines were numerically quite close to each other while scores with the wrap-covered microphones appeared to be higher or lower than the baseline. However, no pattern was immediately apparent. A repeated-measures analysis of variance was calculated with the condition and the speech stimuli (non-nasal, mixed, nasal) as the within-subject effects. The main goal of this analysis was to evaluate possible condition effects. Since these could not be assessed without considering speaker and speech stimulus effects, a relatively complex repeated-measures analysis of variance model resulted. The results showed a subject effect of higher nasalance scores for the female participant. This effect was not unexpected since there were numerous repetitions of the stimuli but only two speakers. The female speaker had higher nasalance scores, which reflects common findings in the literature (see Marino et al., 2015 for an overview). There was no significant effect for condition, which was the main reason the analysis of variance was calculated. The stimulus effect and the pattern of post-hoc comparisons were expected because the speech stimuli were designed to elicit significantly different magnitudes of nasalance (Fletcher, 1978).

While the analysis of variance did not show a significant effect of condition, it would have been imprudent to conclude that the wrap covers did not affect the nasalance scores. The detrimental effect of the wrap covers could be appreciated more clearly from the additional descriptive statistical measures that illustrated the variability of the nasalance scores. The mean and cumulative absolute differences in nasalance between the three repeated recordings of each sound file showed numerically larger variability in nasalance in the wrap cover condition, and smaller variability in nasalance between the two baseline measures. The fact that there were still small numerical differences between the repetitions of the baseline recordings despite the stable recording setup was likely explained by the fact that nasalance scores are reported rounded to the nearest whole number, so minute fluctuations in SPL can result in different rounding solutions. The test-retest differences in the baseline conditions were smaller than what is typically observed in human speakers (De Boer and Bressmann, 2014). The average and added absolute differences between repetitions in the wrap cover conditions were up to 16.6 times larger than between the baselines.

Taken together, the findings showed that wrapping the Nasometer microphone even with something as thin as a layer of household wrap caused marked fluctuations in nasalance scores, within and between wrap cover conditions. In general, SPL measurements at a close range can be affected by small changes in distance, background noise, or calibration of microphones, so Švec and Granqvist (2018) recommended a mouth-to-microphone distance of 30 cm. However, the Nasometer microphones are only approximately 4 cm away from the nose and mouth, and small changes in Nasometer positioning can affect nasalance scores (Watterson and Lewis, 2006). At such a close range, the changes in nasalance scores showed that even the thin Glad Press n’ Seal wrap worked as an acoustic barrier and caused variability within and between wrap cover conditions.

In conclusion, clinicians should not cover Nasometer microphones with Glad Press n’ Seal kitchen wrap as this affects nasalance scores. It is likely that other household wrap products would cause similar distortions. Other materials such as the acoustic foam used for microphone covers may be more suitable but may not provide the level of infection control clinicians might hope to attain. In the present study, face mask material was not considered because it would be much more difficult to fit over the microphones, and materials, design, thickness and absorption properties may differ between different kinds of masks (Corey et al., 2020). Since there is some evidence that the COVID-19 virus can survive for some time on metal surfaces (van Doremalen et al., 2020; Marquès and Domingo, 2021), clinicians should be mindful of the fact that the Nasometer microphone housings can only be cleaned superficially and might best be handled with gloves to minimize any possible risk of touch transfer to the next speaker or the clinician.
